# Rare brain and pulmonary abscesses caused by oral pathogens started with acute gastroenteritis diagnosed by metagenome next-generation sequencing: A case report and literature review

**DOI:** 10.3389/fcimb.2022.949840

**Published:** 2022-09-30

**Authors:** Minhua Chen, Zhiyi Lai, Mingjun Cheng, Tianyu Liang, Zongbin Lin

**Affiliations:** ^1^ Emergency and Critical Care Center, Intensive Care Unit, Zhejiang Provincial People’s Hospital(Affiliated People’s Hospital, Hangzhou Medical College), Hangzhou, China; ^2^ Intensive Care Unit, Ningbo Fourth Hospital (The Affiliated Xiangshan Hospital of Wenzhou Medical University), Ningbo, China; ^3^ Intensive Care Unit, Chun’an First People’s Hospital (Chun’an Branch of Zhejiang Provincial People’s Hospital and Chun’an Hospital Affiliated to Hangzhou Medical College), Hangzhou, China

**Keywords:** brain abscess, odontogenic infection, diagnosis, treatment, metagenomic next generation sequencing

## Abstract

Odontogenic brain and pulmonary abscesses are extremely rare infectious diseases. It is mainly caused by the upward or downward transmission of local infection or blood-borne spread. In recent years, with the wide application of some novel testing methods in clinical practice, the diagnosis of unexplained infections such as odontogenic abscesses in different organs has gradually become clear. We report a case of a 21-year-old male who was healthy and had not received any oral treatment before onset. He started with acute gastroenteritis-related symptoms, then developed meningitis-related symptoms seven days later with septic shock. No obvious abscess lesions were found on head computed tomography (CT) at admission, and the etiology was not clear by routine examination, which was very easy to misdiagnose as a serious infection caused by intestinal pathogens. But odontogenic pathogens were found both in his blood and cerebrospinal fluid through metagenomic next-generation sequencing (mNGS) analysis. Subsequently, rechecked imaging examination displayed multiple brain and pulmonary abscesses. Finally, it was diagnosed as an odontogenic brain and pulmonary abscess. After an extremely lengthy anti-infection course (13 weeks of intravenous antibiotics plus 2 weeks of oral antibiotics) and surgery, the patient was improved and discharged from the hospital. From this case, we could see that the development of new diagnostic technologies such as mNGS plays an important role in the early and confirmed diagnosis of diseases previously difficult to diagnose such as odontogenic polymicrobial infections and ultimately helps to improve the prognosis of these patients.

## Introduction

Brain abscess is a focal infection that originates from locally inflamed areas of the brain parenchyma and develops into a collection of pus surrounded by a well-vascularized capsule (Shruthi et al., 2019). Brain abscesses usually occur in susceptible people, such as patients with immune deficiency, congenital heart disease, or blood-brain barrier injury (Tonon et al., 2006). The incidence of brain abscess of all intracranial masses is around 1% -8% and the 30-day, 90-day, and 1-year mortality rates gradually increased to 7– 11, 13, and 19–20% (Helweg-Larsen et al., 2012; Bodilsen et al., 2020; Gupta et al., 2022). The origin of brain abscesses is usually determined by the presence of acute infectious lesions or definite abscess formation. Imaging findings, such as computed tomography (CT) and magnetic resonance imaging (MRI), usually take a critical role in the clinical examination and diagnosis of brain abscess (Sato et al., 2016). However, in many cases, imaging diagnosis fails to identify pathogenic bacteria, so the treatment of the disease cannot be satisfied. The existing diagnostic technology for abscess bacteria is relatively lacking. We have listed several representative studies in **Table 1**. The ideal treatment strategy should be surgical resection of the abscess and a greater degree of drainage, combined with antibiotics throughout the course of adjuvant therapy  (Antunes et al., 2011). Although few cases of odontogenic brain abscess have been reported, hematogenous dissemination is considered to be the most important pathophysiological mechanism of odontogenic brain abscess (Shibata et al., 2021). Odontogenic brain abscess is one of the brain abscesses that is caused by pathogenic bacteria in the oral cavity entering the brain tissue through blood transmission or upward transmission and is usually seen in a patient with chronic periodontal disease who receive oral therapy (Greenstein et al., 2015). The transmission routes of oral infection to the central nervous system include blood transmission, lymphatic infection, and foreign body infection (Clifton and Kalamchi, 2012). Since oral microbiomes are mostly anaerobic and polymicrobial—making them difficult to culture and identify in routine clinical practice—there are some difficulties in identifying the etiology and taking directed therapy when brain abscess occurs. We report a case of odontogenic brain and pulmonary abscess who was in good health without any medical history of periodontal disease or oral operation.

**Table 1:** Summary of literature related to brain abscess.

Diagnose	Pathogenic bacteria	Detecting techniques	Treatment
Pulmonary actinomycosis with brain abscess (Park et al., 2014)	Actinomyces meyeri	16S ribosomal RNA (16S rRNA) sequencing	penicillin G and metronidazole and oral amoxicillin
Pulmonary abscess、septic embolic cerebral infarction 、cerebral abscess (Albrecht et al., 2012)	Empiric treatment, no bacteria identified	–	piperacillin/tazobactam、ampicillin/sulbactam 、metronidazole
Simultaneous Lung and Brain Abscess (Al-Saffar et al., 2015)	Streptococcus anginosus	brain abscess was biopsied and cultures	vancomycin and ceftriaxone
Lung, Brain, and Spinal Cord Abscesses (Cervera-Hernandez et al., 2021)	Lomentospora prolificans and Scedosporium apiospermum	Sputum culture and DNA sequencing	voriconazole and terbinafine
brain and lung abscesses (Yamada et al., 2016)	Klebsiella pneumonia、Escherichia coli、Streptococcus mitis 、Candida glabrata	abscess culture、blood culture	imipenem/cilastatin sodium、cefotiam hydrochloride、caspofungin
Brain and Lung Abscesses (Browne et al., 2021)	Nocardia cyriacigeorgica	sequence analysis	ceftriaxone, oral trimethoprim-sulfamethoxazole, and a tapering course of dexamethasone.
Brain and Lung Abscesses (Velghe et al., 2004)	Streptococcus milleri	blood culture	penicillin G、clindamycin

## Case presentation

A 20-year-old male patient who was a Zambian student studying in China was transferred from the local hospital to our hospital with the chief complaint of “fever with fatigue for 10 days and unconsciousness for 3 days”. He suffered a high fever (up to 40.8°C) after eating out with his friend 10 days before admission, accompanied by chills, general fatigue, and several cases of vomiting and diarrhea. His friend also had the same symptoms and self-relieved without being diagnosed and treated. But his condition deteriorated, he became unconscious and incontinent, and was sent to the local hospital by his classmates. The routine blood test showed a leucocyte count of 5680 cells/mm^3^ with 90.6% neutrophils, platelet counts of 12000 cells/mm^3^, a C-reactive protein (CRP) concentration of 257.3 mg/L, and a high procalcitonin (PCT) level (>100ng/ml). Suspecting “infectious fever”, blood culture was taken first, and cefoperazone sulbactam was administered intravenously in doses of 2g. He was then transferred to Zhejiang Provincial People’s Hospital, Hangzhou, China on 14 October, 2019, for further treatment.

**Figure 1:**
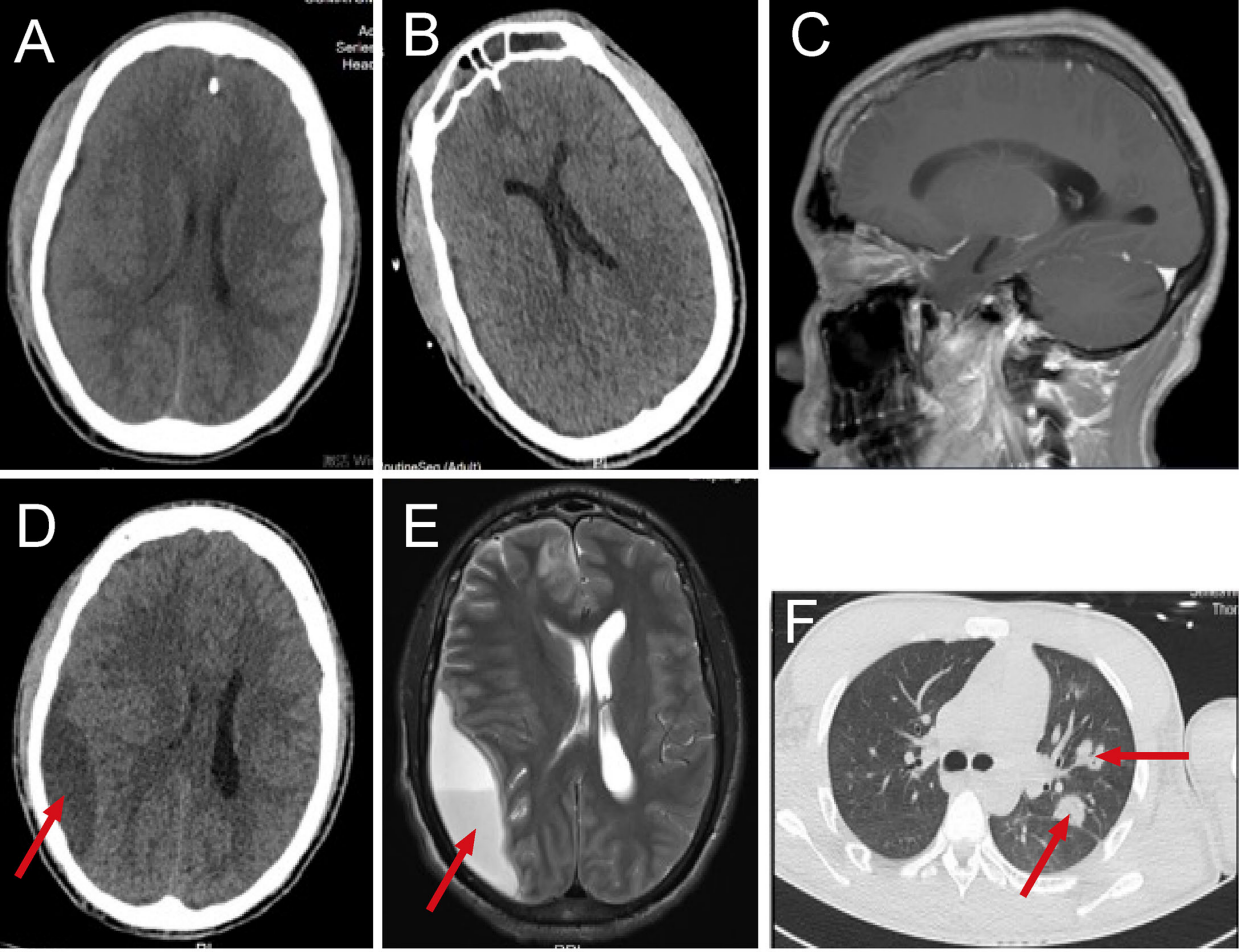
The dynamic imaging examination in the pre-operative period. Head computed tomography(CT) scan at admission **(A)**, head CT on the 7th day of hospitalization **(B)**, the patient’s dental status on magnetic resonance imaging (MRI) **(C)**, head CT on the 18th day of hospitalization **(D)**, head MRI on the 21th day of hospitalization **(E)**, lung CT on the 18th day of hospitalization **(F)**.

His vital signs on admission to our hospital were as follows: temperature, 40.8°C; respiratory rate, 35 breaths/min; pulse, 157 beats/min; blood pressure, 85/42 mmHg. He was intubated because of low oxygen saturation with high flow mask oxygen inhalation. Physical examination showed the patient was in a coma with a Glasgow coma scale (GCS) score of 5 and had a rigid neck. We reexamined his blood and confirmed that the inflammatory markers were significantly increased. Blood tests also found impaired liver and kidney function with an increased level of serum alanine aminotransferase(73 U/L), total bilirubin(206.6 μmol/L), and creatinine(279.2 μmol/L). The results of mNGS in blood and CSF revealed several oral microbiomes, yet we did not find the primary lesion in the patient’s oral cavity. However, we still considered his pulmonary and brain abscesses as odontogenic. There are two reasons for this. First of all, the patient had received anti-infective treatment for a period of time, so the primary oral infection might have been cured. Secondly, studies indicated that there was no definable origin for intracerebral or intraspinal infection in up to 25 % of cases, and they noted that an odontogenic focus should always be considered in the evaluation and treatment of “cryptic” central nervous system infections (Ewald et al., 2006; Akashi et al., 2017). In addition, computed tomography(CT) of the head, chest, and abdomen was taken and revealed a possibly minor subdural effusion in the right fronto-parieto-temporal region, bilateral pulmonary inflammation, and no obvious abnormality in the abdomen (**Figure 1A**). After excluding communicable diseases such as malaria and dengue fever, he was admitted to the intensive care unit(ICU).

After admission, the patient was treated with mechanical ventilation and continuous renal replacement therapy(CRRT). His blood platelet count increased to 50,000 cells/mm^3^ after an emergency platelet infusion of 17U, then the lumbar puncture was dispensed immediately. The cerebrospinal fluid(CSF) was yellow and turbid with a pressure of up to 400mmHg, positive Pandy’s test, low glucose level (1.53 mmol/L), highly elevated leukocytes (1585.8 cells/μL) and protein (>300 mg/dL). According to the results of CSF, intracranial infection was basically clear while the etiology was unknown. Since the patient started with gastrointestinal symptoms, gut-derived intracranial infection was first considered, and intravenous administration of linezolid 1.2g/day combined with meropenem 6.0 g/day was started. On the 3rd day of hospitalization, a blood culture from the local hospital revealed gemella morbillorum, but the cultures of blood, CSF, stool, and other parts that were taken in our hospital were all negative. Therefore, on the 4th day of hospitalization, we performed lumbar puncture again and sent blood and CSF specimens for metagenomic next-generation sequencing (mNGS) in addition to routine culture. The results of blood and CSF culture were still negative, but the results of mNGS in blood and CSF were positive, and a series of oral microbiomes were found (**Table 2**). At the same time, a patchy low-density focus of the right frontal lobe and a minor arc-shaped low-density lesion of the right fronto-parieto-temporal region were seen on rechecked head CT (**Figure 1B**). From the above results, odontogenic intracranial infection was suspected. In terms of treatment, we stopped linezolid and retained meropenem as the targeted antibiotic. After 7 days of treatment, the patient’s condition improved significantly. He became conscious, was weaned from CRRT, and was extubated successfully. However, after inquiring about the medical history, the patient denied having dental caries, periodontitis, or other dental diseases, had no history of oral treatment before the onset, and there was no obvious abnormality in a routine oral examination. CT and MRI showed that the patient’s dental status was basically normal (**Figure 1C**). On the 18th day after admission, the patient had blurred vision. The rechecked head CT showed the original suspicious lesion in the right frontal and fronto-parieto-temporal lobe had become abscess-like, with the right lateral ventricle being pressed and the midline structure was shifted to the left (**Figure 1D**). So, the head magnetic resonance imaging (MRI) was taken and confirmed the formation of multiple brain abscesses (**Figure 1E**). In addition, simultaneous lung CT also revealed the formation of multiple pulmonary abscesses (**Figure 1F**). Combined with the results of mNGS, odontogenic pulmonary and brain abscesses were further confirmed.

**Table 2:** The Results of mNGS in Blood and CSF.

Blood				CSF			
**Genus**	**Sequence Reads**	**Species**	**Sequence Reads**	**Genus**	**Sequence Reads**	**Species**	**Sequence Reads**
Prevotella	156	Prevotella oris	106	Porphyromona	1326	Porphyromonas endodontalis	1309
		Prevotella baroniae	25				
Porphyromona	145	Porphyromonas endodontalis	142	Prevotella	1014	Prevotella oris	704
						Prevotella baroniae	105
Campylobacter	135	Campylobacter rectus	95	Fusobacterium	124	Fusobacterium nucleatum	98
		Campylobacter showae	4				
Streptococcus	11	Streptococcus constellatus	3	Streptococcus	35	Streptococcus constellatus	12
Gemella	5	Gemela morbillorum	5	Gemella	28	Gemella morbillorum	28

**Figure 2:**
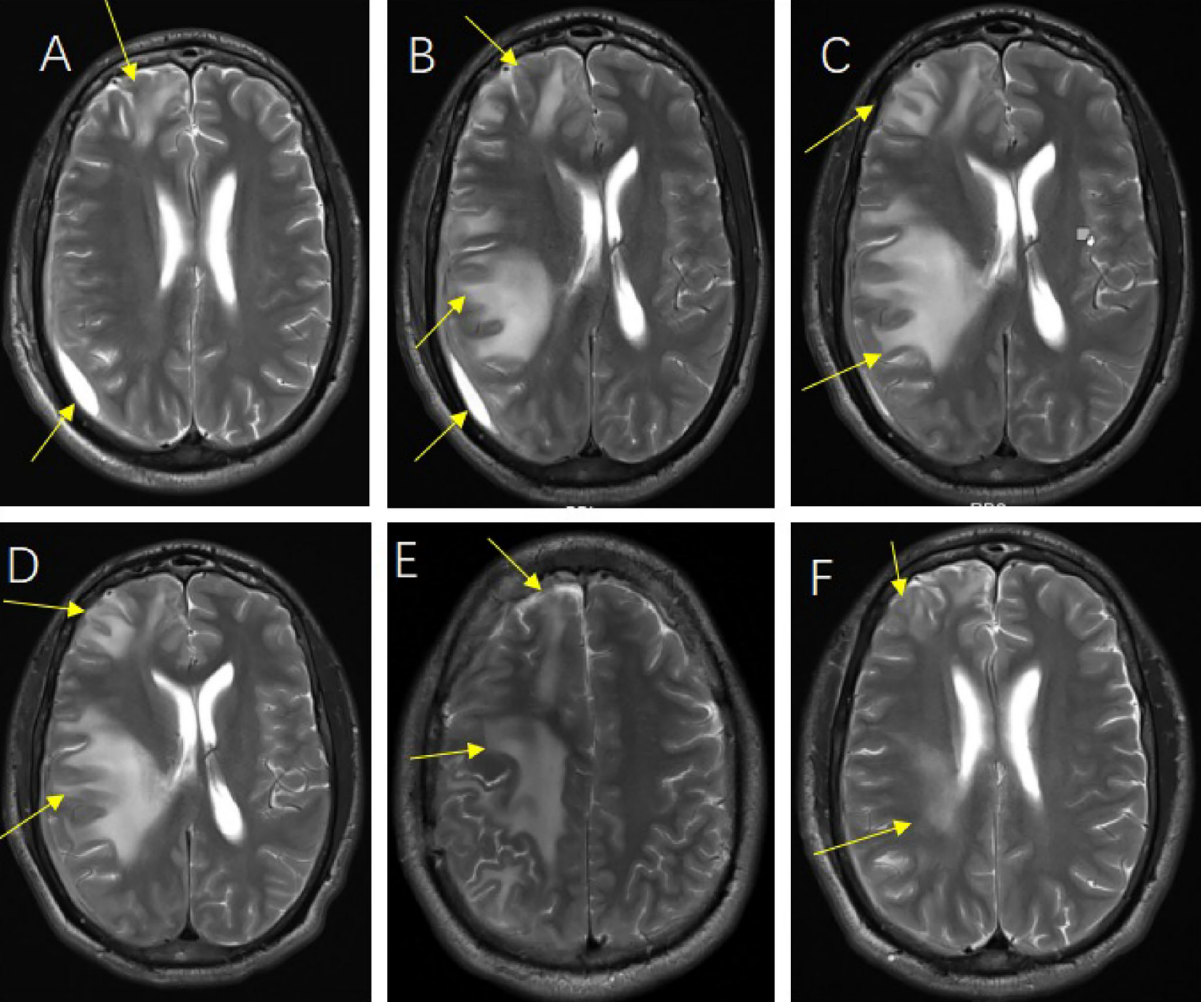
The follow-up head magnetic resonance imaging (MRI) image of post-operation. Head MRI on the 5th day after operation **(A)**, Head MRI on the 14th day after operation **(B)**, Head MRI on the 23rd day after operation **(C)**, Head MRI on the 30th day after operation **(D)**, Head MRI at the time of discharge **(E, F)**.

**Figure 3:**
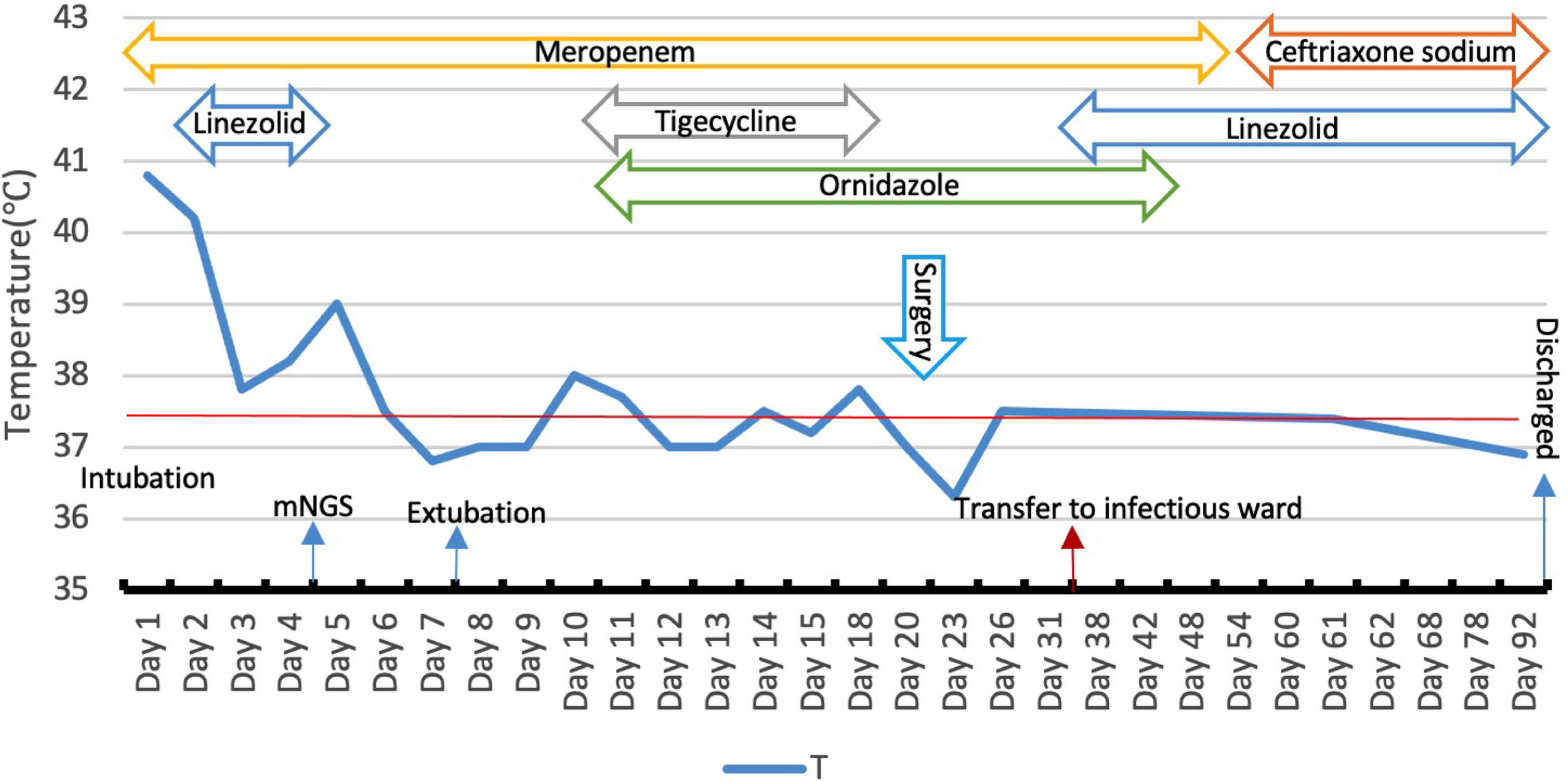
Body temperature and anti-infective treatment during hospitalization, meropenem 2.0 g q8h (day 1–55), linezolid 600mg q12h (day 2–5, day 36–92), tigecyclin 100mg q12h (day 10–15), ornidazole (day 13–54), ceftriaxone(day 56–92).

**Figure 4:**
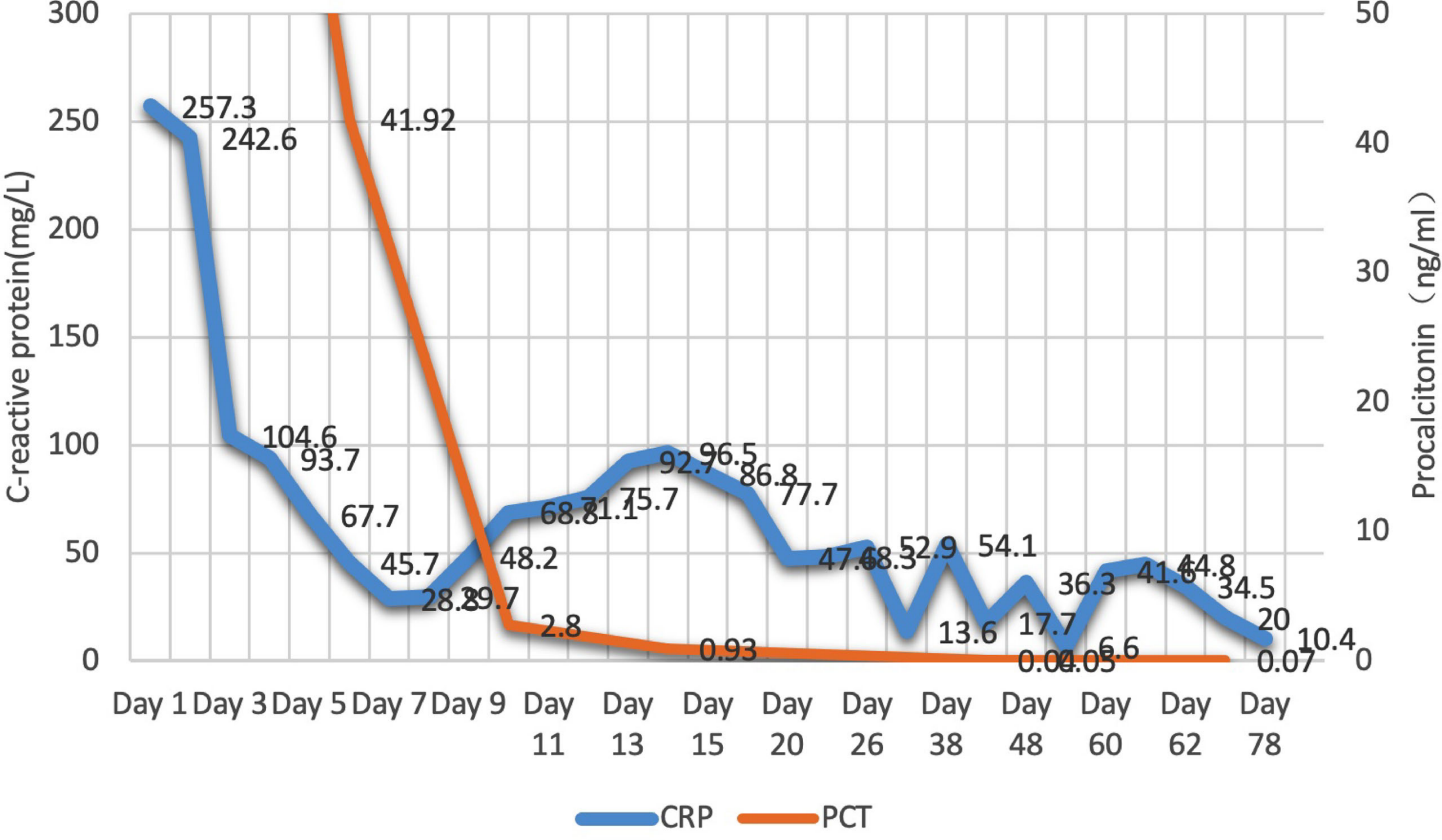
Change in C-reactive protein (CRP) and procalcitonin(PCT) during hospitalization.

For brain abscess, neurosurgery was performed to drain the right subdural abscess, about 40ml of yellow-green pus gushed out and a pus sample was collected during the operation. However, only a culture of the pus was performed, while mNGS was not. Unfortunately, no positive results were obtained from the culture. We speculate that this may be due to the inaccuracy and specificity of routine tests. After the operation, we rechecked his head MRI every 7-10 days. The MRI on the 5th-day post-operation showed the brain abscess was significantly smaller (**Figure 2A**), but worsened in the MRI on the 14th day post-operation (**Figure 2B**). By then, the patient was treated in the intensive care unit for 36 days. Considering the overall stability of the patient’s condition, he was transferred to the infection ward to continue the treatment, meropenem, ceftriaxone, ornidazole, and linezolid was given intravenously successively, and his temperature returned to normal after 9 weeks of anti-infective treatment (**Figure 3**). In accordance with temperature, C-reactive protein (CRP) and procalcitonin (PCT) during hospitalization dropped continuously (**Figure 4**). Unlike the temperature, the follow-up head MRI showed no significant changes of the brain abscesses (**Figures 2C–F**), thus his anti-infective treatment lasted for 92 days’ hospitalization. In addition, the patient had recurrent headaches and seizures during hospitalization, which were improved after the treatment of mannitol and sodium valproate. As the patient strongly requested to be discharged and his general condition was good enough, he was discharged with linezolid tablets for 2 weeks on January 13, 2020. After a follow-up on WeChat 1 year later, he had returned to Zambia and did not present at any hospital for a medical check-up; linezolid was taken orally for 2 weeks and then ceased by the patient, as he deemed he had fully recovered.

## Literature review

The incidence rate of brain abscesses including odontogenic brain abscesses in developed countries is estimated to be 0.3-0.9 per 100,000 people. Brain abscess usually occurs in patients with related susceptibility factors, such as a history of immunosuppressive therapy, craniocerebral surgery or trauma, and congenital heart disease (Corson et al., 2001). For odontogenic brain abscess, it is more common in children or immunosuppressive people, most patients always have acute or chronic oral diseases, poor oral hygiene, or a history of oral therapy (Mameli et al., 2019). It seldom occurs in adults with normal immune status and oral hygiene who also have not received any oral therapy before the onset like in our case. In addition, as our patient started with gastrointestinal symptoms, it was easily misdiagnosed as being caused by intestinal pathogens if there were no pathogenic results. The update from the mNGS results finally helped us to confirm the diagnosis of odontogenic brain and pulmonary abscess. We would be more inclined to consider that the onset of acute gastroenteritis and subsequent multiple brain and pulmonary abscesses were not different stages of the same disease. More than likely, acute gastroenteritis put the patient in a state of low immunity, which created conditions for the invasion of oral microbiome.

The nature of brain abscesses is often polymicrobial, which means conventional culture methods are easily hampered. One recent study confirmed the polymicrobial nature of brain abscesses because NGS helped identify two or more bacterial taxa in 31/51 brain abscesses (Stebner et al., 2021). Another study showed bacterial infections were monomicrobial in 20 cases and polymicrobial in 15 of 41 cases of spontaneous brain abscess, while the microorganisms of the polymicrobial cases were restricted to the oral microbiome. The oral microbiome is mostly anaerobic and polymicrobial, which may not be detected by conventional culture methods, either untargeted therapy or directed therapy toward a single detected bacterium could be problematic (Andersen et al., 2022). However, mNGS is not a targeted diagnostic method, but a technology based on the principle of metagenomics. It obtains the species information of suspected pathogenic microorganisms through extracting the nucleic acid from the samples, using conventional library construction process and the second-generation high-throughput sequencing platform for nucleic acid sequencing, then analyzing with the special microbial database. Theoretically, mNGS can identify almost all microorganisms simultaneously as long as the reads are long enough with multiple hits in the microbial genome, and the reference database is large enough (Miller et al., 2019). Moreover, as the technology improves, the time required for sequencing has been greatly reduced, which provides patients with a more accurate treatment strategy (Li et al., 2021). So mNGS can overcome the limitations of conventional culture methods with higher sensitivity and faster speed in pathogen identification and is less affected by antibiotics. mNGS has been successfully applied to the detection of the pathogen in CSF, and its application is becoming more and more common. A large prospective multicenter study of 213 patients has demonstrated that mNGS was more effective and rapid for identifying pathogens causing infectious central nervous system diseases than conventional microbiological testing (Xing et al., 2020). A systematic review showed there was increasing evidence of a role for NGS in the work-up of undiagnosed encephalitis by identifying 25 articles reporting 44 case reports of patients with suspected encephalitis for whom NGS was used as a diagnostic tool (Brown et al., 2018). Furthermore, other studies also confirmed NGS methods not only expanded the spectrum of pathogens detected, but also increased sensitivity without losing specificity in the etiology identification of brain abscesses (Stebner et al., 2021). Additionally, it had been proved that 40-60% of oral microbiomes were as-yet-uncultivated, the culture of the oral microbiome was often prone to errors and sometimes did not result in any bacterial growth (Siqueira and Rocas, 2013; Bottger et al., 2021a). Studies also confirmed the advantages of NGS in detecting oral microbiome, with a higher number of bacteria and a significantly higher proportion of anaerobes (Bottger et al., 2021b).

Secondly, pulmonary cavitation attributable to infection by oral microbiome has been well described before (Guo et al., 2019). But up to the present, few cases of odontogenic brain and pulmonary abscess have been reported. Hughes et al. reported a case of a suspiciously odontogenic orbital abscess, cavitatory pulmonary disease, and meningitis because the oral microbiome was cultured from the orbital abscess. Like in our case, they did not get positive culture results from a respiratory tract specimen. The recovery of the patient’s cavitatory pulmonary lesions and aseptic meningitis after intravenous antibiotics strongly supports an infectious etiology (Hughes et al., 2017). In addition, the route and mechanism of oral microbiome invading the brain and lung is unknown and needs to be clarified. Studies have shown that the formation of brain abscesses can be transmitted by blood and from adjacent infected sites, and blood transfer is the main mode of transmission (Muzumdar et al., 2011; Moazzam et al., 2015). As we know, periodontium provides a good living environment for microorganisms due to its special anatomical characteristics. So oral operation and improper oral care (even daily brushing) may cause local infection and become the source of bacteremia. Repeated bacteremia begins to invade the whole body when the body’s immunity is impaired, thus the brain and lung are inevitably infringed (Lockhart et al., 2008).

Finally, when it comes to the treatment, the recommended duration of intravenous antibacterial treatment for patients with bacterial brain abscesses is 6-8 weeks (Brouwer and van de Beek, 2017). A retrospective review of adult patients with pyogenic brain abscesses showed the median duration of antibiotic treatment was 62 days (Helweg-Larsen et al., 2012). Generally, the course of antibiotics usually depends on the evaluation of clinical efficacy including the temperature, patient’s neurological symptoms, and imaging changes of abscess (Brouwer et al., 2014). The case we reported above not only underwent abscess drainage but also had a super long period of anti-infective treatment which lasted nearly 13 weeks intravenously plus 2 weeks orally due to dynamic MRI reexamination showed that the absorption of brain abscesses was very slow, and neurological symptoms recured while the general condition was improved quickly. It also took a long time for his temperature to return to normal. The patient’s brain abscess was not fully absorbed at discharge, but he stopped taking medicine after discharge on no authority but his own. Fortunately, it seems he indeed fully recovered as was his own judgment without any medical reexamination. The treatment process of this patient may suggest that the length of antibiotic treatment for brain abscess should refer to clinical manifestations rather than the imaging regression state of brain abscess.

## Discussion

This paper reports a case of brain abscesses caused by oral bacteria and the treatment effect is satisfactory. The patient had a high fever and was not treated properly at the beginning. After his condition deteriorated, he was admitted to a local hospital for treatment, but the source of the infection was not clear, and he was treated empirically with cefoperazone sulbactam. He was transferred to our hospital with poor treatment. After admission to our department, the patient’s symptoms were not relieved. Tracheal intubation and CRRT support were urgently performed for a patient with persistent high fever and even multiple organ damage. All conventional testing methods were tried but no specific source of bacteria was identified. We empirically diagnosed enterogenous intracranial infection because of the prominent gastrointestinal symptoms. The intravenous administration of linezolid 1.2g/day combined with meropenem 6.0 g/day was started. mNGS testing later revealed that the bacteria were of oral origin, but no definite oral lesions were found, and imaging also revealed intracranial lesions, so linezolid was discontinued. After that, the patient’s symptoms improved significantly, the function of each organ gradually recovered, and the tracheal intubation and CRRT were removed. Unfortunately, the patient’s condition changed, and radiographic evidence revealed the formation of an abscess in the brain. We consulted the neurosurgery department for puncture and drainage. Meropenem, ceftriaxone, ornidazole, and linezolid were given intravenously successively. A series of treatments after the diagnosis was confirmed, the symptoms of the patient improved, the indicators tended to be normal, and the patient was discharged.

The incidence of odontogenic brain and pulmonary abscesses is very low for healthy adults without oral operation or oral diseases. The formation of odontogenic brain abscesses can be transmitted by blood through odontogenic bacteremia or from adjacent infected sites, while pulmonary abscesses may be caused by aspiration or blood transmission. Due to the aggregation of the oral microbiome, polymicrobial brain abscess is a challenge for the clinical microbiology laboratory. Since polymicrobial infections may not be detected by conventional culture methods, targeted treatment might be a big problem at this point, new detection technologies such as mNGS may greatly contribute to the diagnosis and effective antibiotic treatment of the disease, and ultimately improve the prognosis of these patients. To summarize, when clinicians encounter such a patient whose initial symptoms and signs are not related to the nervous system and lack high-risk factors, it poses a challenge to form a correct diagnosis and treatment. At this time, new emerging pathogen detection technologies such as mNGS may provide great help besides routine culture.

## Author contributions

MHC designed experiments. The preliminary preparation work and data collection were carried out by ZBL and MJC. ZYL wrote the manuscript. TYL helped with revision. All authors contributed to the article and approved the submitted version.

## Funding

This work was supported by the General Project Funds from the Health Department of Zhejiang Province (Grant No. 2018KY269).

## Conflict of interest

The authors declare that the research was conducted in the absence of any commercial or financial relationships that could be construed as a potential conflict of interest.

## Publisher’s note

All claims expressed in this article are solely those of the authors and do not necessarily represent those of their affiliated organizations, or those of the publisher, the editors and the reviewers. Any product that may be evaluated in this article, or claim that may be made by its manufacturer, is not guaranteed or endorsed by the publisher.
